# Chemical Fingerprinting, Anti‐Inflammatory, and Antioxidant Potential of Hydroethanolic Extract of *Aesculus indica*

**DOI:** 10.1002/fsn3.4721

**Published:** 2025-02-03

**Authors:** Hina Fatima, Muhammad Shahid, Sana Fatima, Paul J. Mills, Chris Pruitt, Meredith A. Pung, Muhammad Riaz, Rizwan Ashraf, Quzi Sharmin Akter

**Affiliations:** ^1^ State Key Laboratory of Food Science and Resources Nanchang University Nanchang China; ^2^ Department of Biochemistry University of Agriculture Faisalabad Pakistan; ^3^ Herbert Wertheim School of Public Health and Human Longevity Science University of California San Diego California USA; ^4^ Department of Chemistry University of Agriculture Faisalabad Pakistan; ^5^ Department of Allied Health Sciences University of Sargodha Sargodha Pakistan; ^6^ Department of Genetics and Animal Breeding Faculty of Animal Science and Veterinary Medicine, Patuakhali Science and Technology University Patuakhali Bangladesh

**Keywords:** anti‐inflammatory, histopathology, inflammatory mediators, MTT, oxidative stress markers, proinflammatory cytokines

## Abstract

*Aesculus indica* is a remarkable species from Sapindaceae family, traditionally used for the treatment of various ailments due to the presence of a variety of bioactive compounds. The present study was planned to evaluate the chemical characterization, anti‐inflammatory and antioxidant potential of hydroethanolic extract of *A. indica* using in vitro and in vivo approaches. *A. indica* fruit was extracted with a hydroethanolic (70% v/v) solution, filtered, concentrated on a rotary evaporator and crude extract was obtained. In vitro anti‐inflammatory potential of *A. indica* was carried out against peripheral blood mononuclear cells (PBMCs) and a whole blood assay (WBA). Effects of *A. indica* extracts on proinflammatory cytokines (TNF‐α, IFN‐gamma, IL‐6, IL‐1β) and inflammatory mediators (NF‐κB, NO and PGE_2_) concentration in the supernatant of PBMCs and WBA were evaluated using commercial ELISA kits. In vivo anti‐inflammatory potential of *A. indica* hydroethanolic extract was evaluated with carrageenan‐induced paw edema in rats. A total of 36 different compounds (mostly phenolics) were detected in *A. indica* extract with high performance liquid chromatography (HPLC) and UHPCL‐QTOF‐MS/MS. The extract showed very low cytotoxicity with an IC_50_ value of 483.68 μg/mL and significantly reduced the levels of proinflammatory cytokines and inflammatory mediators in both PBMCs and WBA models. Furthermore, the extract also effectively inhibited the paw edema by carrageenan in the 2nd hour at 400 mg/kg (73%). Histopathological analysis of rat paw tissue showed significant reduction of cellular infiltration and decrease in swelling of epidermis and dermis by *A. indica* extracts. The level of enzymatic antioxidants such as superoxide dismutase (SOD) and Catalase (CAT), lipid peroxidation like malondialdehyde (MDA), oxidative stress parameters including total antioxidant status (TAS) and total oxidant status (TOS) and myeloperoxidase (MPO) activity in rat paw tissues were significantly altered after treatment. The combined findings provide evidence that hydroethanolic extract of *A. indica* is a potential source of bioactive compounds with significant anti‐inflammatory and antioxidant activities.

## Graphical abstract

*Aesculus indica* extract reduces cellular infiltration, and other inflammatory parameters related to oxidative stress in carrageenan‐induced rat paw edema model. *A. indica* is a potential source of bioactive compounds with significant anti‐inflammatory and antioxidant activities.

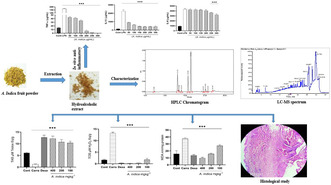

## 1 Introduction

Nature offers a wide range of herbs for the treatment of various diseases of mankind (Shah et al. 2019; Zaman et al. 2022). Since ancient times, traditional medicinal plants have been used in several countries for the treatment of various ailments due to their cost effectiveness and low toxicity. Therefore, medicinal plants have gained great attention in the development of herbal medicines, attributed to their phytoconstituents (Kauser et al. 2018; Vitale et al. 2022). The WHO reports showed that more than 80% of people worldwide depends mainly on medicinal plants for basic health care (Riaz, Abbas, et al. 2023; Tao et al. 2022). Natural products have contributed greatly to the progress of modern medicine (Saad, Kmail, and Haq 2022; Sharif et al. 2018). Pharmaceutical markets such as analgesics, anti‐inflammatory, anticancer agents and antibiotics have been discovered as a result of centuries of searching for new therapeutic agents from natural resources (Jambwa et al. 2022; Shahid et al. 2022). The derivatives of medicinal plants are in great demand worldwide as first‐line treatments for human health. Originally, these plants were used by humans in their raw form (in the form of tea, tinctures, powders, etc.), but successful isolation of codeine, cocaine, and quinine opened a new era of herbal medicine. Further exploration in this area turned to successful isolation of morphine from opium which was a breakthrough (Anand et al. 2022; Farooq et al. 2022; Riaz, Khalid, et al. 2023).

The family Sapindaceae (subfamily Hippocastanaceae) consists of 138 genera and 1858 species including horse chestnut. *Aesculus indica*, also known as Himalayan chestnut or Indian horse chestnut is found in temperate regions of Asia, America, and Europe (Yadav et al. 2022). In Asia it is generally found in Pakistan, Nepal and India. It is traditionally used for the treatment of skin diseases, rheumatism, diabetes, hemorrhoids, phlebitis, thrombosis and abdominal colic (Faisal et al. 2022). The plant reported to contains aescin, rutin, quercitrin, mandelic acid, β‐sitosterol, astragalin and several other bioactive compounds. It is extensively used in traditional medicines owing to its medicinal benefits. Roots, bark and seeds of *A. indica* possess anti‐rheumatic properties; fruits are utilized against diabetes and colic diseases and leaves are known for anti‐cancerous activities. It is very useful for hemorrhoids, ulcers, varicose veins and help against thrombosis. The plant is also effective in the treatment of frostbite, bruises and migraines (Riaz et al. 2022; Zahoor et al. 2018).

Inflammation is part of a complex defense mechanism of the body to noxious stimuli such as harmful biological or chemical agents (Bisgaard et al. 2022). Inflammation triggers the release of several hydrolytic enzymes, exudation of fluid, vasodilation, increase in vascular permeability and blood pressure, destruction, and repair of injured tissue. Inflammatory response leads to activation of different immune cells in the body which produce free radicals (NO and ROS) causing peroxidation of lipids and localized tissue damage (Yuan et al. 2023). Damage caused by free radicals contributes to the pathogenicity and the development of oxidant stress associated diseases such as inflammatory diseases. Several anti‐inflammatory drugs are recommended for symptomatic relief of different inflammatory conditions such as toothache, sore throat, muscle pain, fever and arthritis (Phull, Ahmed, and Park 2022). The prolonged use of anti‐inflammatory drugs both topically (sprays, creams and gels) and orally may have serious side effects. Therefore, natural medicines with less side effects, more efficacy and proven safety are essential for the substitution of chemical agents (Toenders et al. 2022). Medicinal plants with proven pharmacological activities in traditional medicines as anti‐inflammatory and antioxidant agents may provide as significant source of natural remedies against inflammatory diseases (Qomaladewi et al. 2019).

In this research study, we assessed the chemical characterization and anti‐inflammatory potential of *A. indica* through different assays. To our knowledge, no scientific reports are available on the anti‐inflammatory potential of *A. indica* in vitro against these models. Further, the data on chemical characterization of this plant is also very scarce. Therefore, we planned experiments to assess the metabolite profiling and anti‐inflammatory potential of *A. indica* in acute inflammation, using different experimental models. The preliminary investigations included an acute toxicity test. Inhibition of prostaglandins (PGE2), nitric oxide (NO), and proinflammatory cytokine (TNF‐α, IL‐6, IL‐1β, IFN‐γ) release from PBMCs and WBA are used as markers for evaluation of anti‐inflammatory potential.

## 2 Materials and Methods

### 2.1 Preparation of Plant Extract

The fruit of *A. indica* was collected from the hilly areas of Abbottabad, Pakistan. This plant is well known by its local name Bankhor. For authentication, plant material was submitted to the Department of Botany, University of Agriculture, Faisalabad. Collected parts (fruit) of plant were washed with distilled water and shade dried. The dried plant material was thoroughly grounded and passed through a 100 mm sieve to make homogeneous fine powder. Plant material was extracted with 70% ethanol and water mixture under constant shaking for 3 days. Solvent mixture was filtered and produced a concentrated material under reduced pressure (Fatima et al. 2021).

### 2.2 Chemical Characterization of *A. indica* Extract

#### 2.2.1 HPLC‐PDA Analysis

Phytochemical profile of *A. indica* extract was explored through high performance liquid chromatography (HPLC) of model (waters alliance 2998) WATERS USA equipped with photodiode array detector (PDA). The analytical performance was achieved through adjusting composition of mobile phase, temperature of column, chemistry of column as well as flow rate of mobile phase (Hasany et al. 2020). A reverse‐phased octadecylsilasil column (Spherisorb ODS‐2) having particle size 10 μm, and dimensions (300 × 4.6 mm) was used for elution under gradient programing of mobile phase solution A (0.5% acetic acid) and solution B (Methanol). Gradient programming was maintained as 90A:10B (0–4 min), 80A:20B (4–8 min), 60A:40B (8–16 min) and 50A:50B (16–25 min) and again equilibrate at initial conditions for further 5 min with constant flow rate of 1 mL/min, and chromatograms along with corresponding spectrums at 400‐200 nm were acquired through Empower 3.0 software. For quantitative analysis, stock solution (0.5 mg/mL) of individual standards were prepared in methanol and preserved. Calibration solutions were prepared from stock solution at concentrations including 1–50 ppm (1, 10, 20, 30, 40 and 50 ppm) in Methanol. Samples were prepared with fresh weight 1 mg/mL in Methanol in triplicate.

#### 2.2.2 LC–MS/MS Analysis

Detailed phytochemical profile of *A. indica* was studied through Quadrupole time of flight mass detector (QTOF‐MS/MS) chromatographed through ultrahigh performance liquid chromatography (UHPLC) model 6520 Accurate‐Mass Q‐TOF LC/MS Systems of make Agilent Technologies Santa Clara, California, United States. UHPLC separation conditions were optimized as through gradient programming of mobile phase with solution A (0.1% formic acid) and solution B (0.1% formic acid in Methanol) of which gradient flow starts as 15%–30% A at time 1–15 min, 30%–40% A at 15–25 min, 40%–50% A at 25–35 min and 50%–10% A at 35–40 min while constant flow rate of 0.4 mL/min. Electrospray ionization (ESI) was used as ionization source operating at both modes, that is, negative and positive ion mode while nitrogen gas was used as collision gas. Nebulizer pressure of ionization source was adjusted at 35 psi while capillary temperature was maintained 350°C. Other parameters of Ion source like, drying gas flow rate was at 10 L/min, octapole RF peak voltages, VCap, fragmentor and skimmer were maintained to 740, 3500, 150 and 65 V, respectively, and samples were scanned in the range of 150–1200 Da. Each peak was characterized for MS/MS fragmentation pattern of selected precursor ions (Zhang et al. 2018).

### 2.3 Ethics Statement

The PBMC and WBA in vitro studies were performed according to the principles of the Declaration of Helsinki and were approved by the responsible ethics committee (Ethics committee of the University of California, San Diego, USA). Human blood was taken from healthy volunteers (*n* = 10) after informed written consent and agreement about buffy coats that would be used for research purposes only. The in vivo animal portion of the study was approved by the ethics committee IACUC (Institutional Animal Care and Use Committee) of University of Agriculture Faisalabad, Pakistan.

### 2.4 PBMCs Isolation by Standard Gradient Technique

Fresh blood from volunteers was used to separate PBMCs through standard Ficoll‐Paque centrifugation method. In brief, this process was executed through 4 mL of Ficoll‐Paque reagent that was transferred in Falcon tubes (15‐mL centrifuge tubes). After centrifugation process, heparinized blood was further diluted with phosphate‐buffered saline (1:1) and accurately layered with Ficoll‐Paque reagent (~10 mL). This solution was centrifuged at 900 *g* for 20 min while temperature maintained at 20°C. The resultant solution was harvested carefully for cell interface layer and cells were washed twice with phosphate buffer saline and this solution was further centrifuged at 640 *g* for 10 min followed by centrifugation at 470 *g* for 10 min. Resultant product was resuspended in RPMI 1640 medium augmented with streptomycin (100 μg/mL), Fetal bovine serum (10% FBS) and 100 IU/mL penicillin before cell counting. Cells were counted by hemocytometer using Trypan blue exclusion method. Cells were resuspended in RPMI (Roswell Park Memorial Institute Medium) and used for PBMCs stimulation assay (de Lima et al. 2019).

### 2.5 Cell Viability Assay

All the studied samples were processed for cell viability assay through well‐known MTT assay. For PBMCs, 1 × 10^5^ cells at a density of 2 × 10^4^ cells were incubated in 96 well microtiter plate and incubated with increasing concentrations (100–1000 μg/mL) of *A. indica* extract in triplicates and incubated for 4 days at 37°C and 5% CO_2_. In this experiment, Doxorubicin (100 μg/mL) was used as standard. After initial incubation, MTT solution (20 μL, 5 mg/mL) was added into wells and again incubated for 4 h under the same conditions. Following the second incubation, the plates were centrifuged for 20 min at 800 *g*. Supernatant was discarded and DMSO (100 μL) was added into each well to dissolve the formazan crystals (de Lima et al. 2019). Cell viability (%) was calculated relative to negative control by reading the plate at 570 nm and IC_50_ value was calculated from GraphPad prism (GraphPad Software Inc. CA, USA).

### 2.6 PBMCs Stimulation Assay

Isolated PBMCs (1 × 10^5^ per well) were incubated with different concentrations of *A. indica* extract (50–300 μg/mL), Lipopolysaccharide (LPS; 5 μg/mL), and Dexamethasone under 5% CO_2_ at 37°C for 24 h. After incubation the cells were centrifuged at 500 *g* for 10 min. Supernatant was then collected and stored at −80°C until analysis (Fatima et al. 2022; de Lima et al. 2019).

### 2.7 Whole Blood Assay

Heparinized whole venous blood was collected in lithium heparin containing tubes and diluted five‐fold with RPMI 1640 medium (serum‐free) augmented with penicillin (100 IU/mL), and streptomycin (100 μg), and processed within 2 h of collection. Diluted blood (100 μL) was incubated with different concentration of plant extract (50–300 μg/mL) and LPS (5 μg/mL) was added to each well of a round bottom tissue culture treated plate. Dexamethasone was used as standard. Cultures were incubated for 24 h at 37°C at 5% O_2_. After 24 h, plates were centrifuged at 500 *g* for 10 min at 20°C and supernatants was stored at −80°C until analysis (Whatney et al. 2018). For measurements of PGE_2_, Indomethacin was added into blood at a concentration of 10 μg/mL after collection to inhibit the conversion of arachidonic acid to PGs (Prostaglandins).

### 2.8 Human Proinflammatory Cytokines

The levels of TNF‐α, IFN‐γ, IL‐1β and IL‐6 were measured using an electrochemical‐luminescence‐based sandwich immunoassay method (multi‐array)using the MSD Human Proinflammatory Panel‐1 V‐PLEX 10‐spot multiplex kit (Meso Scale Diagnostics LLC, Rockville, MD) (Dabitao et al. 2011).

### 2.9 NF‐Kappa‐B Assay

The NF‐Kappa‐B (NFκB1) was measured using a quantitative enzyme immunoassay method through a NFκB1/NF‐κ‐B ELISA kit (LifeSpan Biosciences Inc., Seattle, WA) (Lee et al. 2012).

### 2.10 Measurement of NO and PGE2

The total nitric oxide and nitrate/nitrite and prostaglandin E2 (PGE2) were measured using a quantitative enzyme immunoassay method through R&D Systems Parameters ELISA kit (Bio‐techne, Minneapolis, MN) (Elias et al. 2019).

### 2.11 In Vivo Assay

#### 2.11.1 Animals

Healthy Wistar rats of about 150–200 g of both the gender were used in the in vivo assays. Animals were keptin polypropylene cages in dark and light cycle (12 h light/dark) by maintaining temperature 24°C ± 2°C, and 40%–60% humidity. Rodent food and water were freely available for rats during the entire period. However, rats were kept deprived from food but not from water just 4 h before the experiment (Naz et al. 2020).

#### 2.11.2 Acute and Sub‐Acute Toxicological Assessment of *A. indica*

For acute toxicity study, rats were grouped into two sets (*n* = 6), with one set served as control while the other as treatment. The *A. indica* extract was given through oral gavage with a dose of 2000 mg/kg of rat weight to the treatment set that had previously been fasting for 7–8 h. The rats were treated with vehicle (70% ethanol/water) and *A. indica* extract only once (day 0) at the beginning of the experiment and rats were monitored for 14 days. For subacute study, animals were placed into 4 groups (*n* = 6). Group 1 receive vehicle (70% ethanol/water mixture) while Group 2, 3 and 4 received 250, 500 and 1000 mg/kg of *A. indica* extract daily for 28 days. On 28th day, final weight of the animals was measured, and then anesthetized under diethyl ether to obtain blood samples from each rat individually by cardiac puncture. After blood samples collection, all the rats were sacrificed to take their liver and the kidney parts and these parts were further dissected out for detailed pathological observation to check for any possible overt lesions (Fatima et al. 2021; Olayode, Daniyan, and Olayiwola 2020).

#### 2.11.3 Effect of *A. indica* Extract on Hematological and Biochemical Parameters

Effect of *A. indica* extract on different hematological and biochemical parameters was evaluated. Hematological profile was measured through an automated hematology analyzer while semi‐automated chemistry analyzer was used for the determination of biochemical parameters from serum (Naz et al. 2020; Olayode, Daniyan, and Olayiwola 2020).

### 2.12 In Vivo Anti‐Inflammatory Activity of *A. indica*

#### 2.12.1 Rat Paw Edema Model

The rats were placed into six groups (*n* = 6) and given different treatments as follows.

Group I (vehicle group) rats were treated with 70% v/v ethanol orally. Group II (Disease control group) rats were given carrageenan solution (0.1%) subcutaneously in their right paw. Group III (Reference standard group) rats were given dexamethasone (20 mg/kg of body weight). Group IV (low dose group) and was orally pre‐treated with *A. indica* extract at 100 mg/kg body weight. Group V (Medium dose group) and was orally pre‐treated with *A. indica* at 200 mg/kg body weight. Group VI (High dose group) and was orally pre‐treated with *A. indica* extract at 400 mg/kg body weight. Inflammation was induced with carrageenan solution (0.1%) in the sub‐plantar region of the right paw of each animal exactly after 1 h of *A. indica* extract treatment. The volume of rat paw was noted at different intervals of 0, 1, 2, 3, 4 and 5th hours after carrageenan injection. The percentage inhibition (PI) was calculated against vehicle group (Fatima et al. 2021; Rafiee, Hajhashemi, and Javanmard 2019).

#### 2.12.2 Histopathological Examination of Rat Paw

After 5th hours of carrageenan injection, the rat paw tissues were fixed in formalin solution (10%) for 72 h and then decalcified with formic acid (10%) solution. The paw tissues were then dehydrated with increasing concentrations of alcohol, paraffin embedded and cut into sections of 5 μm thickness. The prepared tissues were stained using hematoxylin and eosin staining procedure and the infiltration of inflammatory cells was evaluated (Uroos et al. 2017).

#### 2.12.3 Enzyme Assay and Oxidative Stress Markers

Samples from rat paw tissues were homogenized in 50 mM HEPES (N‐2‐Hydroxyethylpiperazine‐NV‐2‐ethanesulfonicacid) and 0.2 mM PMSF (phenylmethylsulfonyl fluoride buffer). The prepared homogenate was then centrifuged at 800 *g* for 20 min. The supernatant was collected and again centrifuged 5000 *g* for 15–20 min (Du et al. 2018). The effect of *A. indica* extract on MPO, MDA, SOD activity, CAT activity, TOS and TAS in paw tissue homogenate was measured (Baliga et al. 2018; Ben Khedir et al. 2016; Riaz et al. 2016; Wu et al. 2017).

### 2.13 Statistical Analysis

All experiments were conducted in triplets, and obtained data was expressed as mean and standard deviation of means. One‐way analysis of variance (ANOVA) combined with Dunnett's multiple comparisons test were conducted using GraphPad Prism Version 7.0 for Windows (GraphPad Software, San Diego, USA) (Johnson 2014).

## 3 Results and Discussion

The concept of herbal medication is becoming increasingly popular and the demand for herbal products continues due to their efficacy, cost effectiveness and perhaps desire for more natural treatments with relatively fewer side effects (Vitale et al. 2022). Natural products are a copious source of bioactive compounds that can be developed as therapeutic drug candidates and beneficial dietary supplements (Anand et al. 2022). The current project was aimed to check the anti‐inflammatory activity of *A. indica* extract with different in vitro and in vivo methods.

### 3.1 Chemical Characterization of *A. indica* Extracts

#### 3.1.1 Phytochemical Screening Through HPLC‐DAD

**FIGURE 1:**
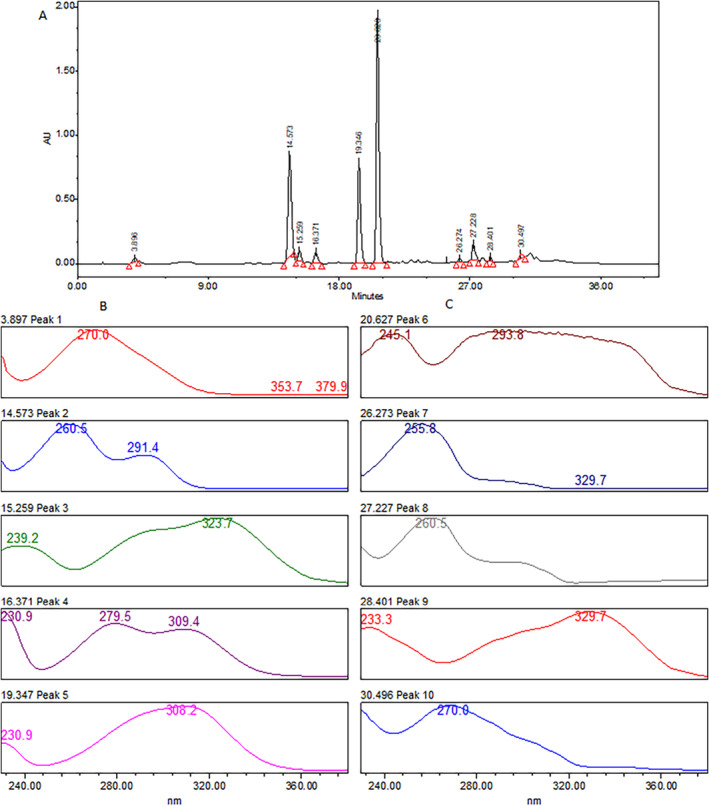
(A) HPLC‐PDA chromatogram of *Aesculus indica* extract (B) UV–Visible spectra of peak 1‐peak 5 of HPLC chromatogram, (C) UV–Visible spectra of peak 6‐peak 10 of HPLC chromatogram.

Phytochemical screening of *A. indica* extract was made through high performance liquid chromatography provided with photodiode array detector using chromatographic conditions discussed earlier and chromatogram is presented in Figure 1. Almost 10 phytochemicals were identified through HPLC‐DAD which were confirmed through standard material as well as UV–Visible spectrum of each peak and results are given in Table 1. Among the identified phytochemicals, most of the compounds were phenolics like gallic acid, coumaric acid etc. and some of them were identified flavons when compared with UV‐spectrum of NIST library. The first tiny peak in the chromatogram of HPLC was observed at retention time of 3.89 min which showed lambda maximum at 270.0 nm and identified as gallic acid. The prominent peak was observed at 14.53 min having UV spectral behavior similar to trihydroxybenzoic acid which further confirmed through standard and identified. The other phytochemicals were also identified in the similar pattern and confirmed through NIST library, results are presented in Table 1. The identified phytochemicals were quantified, and results are presented in terms of milligram of active constituent per 100 g of fresh weight of sample. Among the identified phytochemicals, p‐coumaric acid was found in highest quantity of 234.6 ± 2.1 mg/100g FW. The other compounds were also in significant quantity and lies in the range of 36.7–117.5 mg/100g FW. Our findings were in line agreement with published reports which reported the presence of higher amount of p‐coumaric acid in *A. indica* extracts (Zahoor et al. 2018). The biological properties of *A. indica* extract may be attributed due to these phytochemicals. Interestingly all the phytochemicals detected in the sample have hydroxyl groups that might be related for antioxidant potential of studied sample. Obtained peaks of HPLC were recognized by comparing with standards and UV–visible match index of each peak through NIST library.

**TABLE 1:** Summary results of phytochemicals quantification of *Aesculus indica* extract estimated through HPLC‐PDA.

Peak number	Retention time	Compound name	Lambda maximum (nm)	Concentration mg/100 g FW
1	3.89	Gallic acid	270.0	97.5 ± 1.1
2	14.57	Trihydroxybenzoic acid	260.5, 291.4	107.6 ± 0.8
3	15.25	p‐hydroxybenzoic acid	239.2, 323.7	117.5 ± 1.1
4	16.37	Vanillic acid	230.9, 279.5	58.63 ± 0.5
5	19.34	p‐coumaric acid	230.9, 308.2	234.6 ± 2.1
6	20.62	Carnosic acid	245.1, 293.8	36.7 ± 0.3
7	26.27	Quercetin	255.8	157.8 ± 1.2
8	27.22	Apigenic acid	260.5, 304.6	111.9 ± 1.8
9	28.40	Ferulic acid	233.3, 329.7	126.4 ± 1.4
10	30.49	3‐Chlorogenic acid	270.0	69.8 ± 0.8

#### 3.1.2 Phytochemical Screening Through LC–MS/MS (ESI‐Q‐TOF)

**FIGURE 2:**
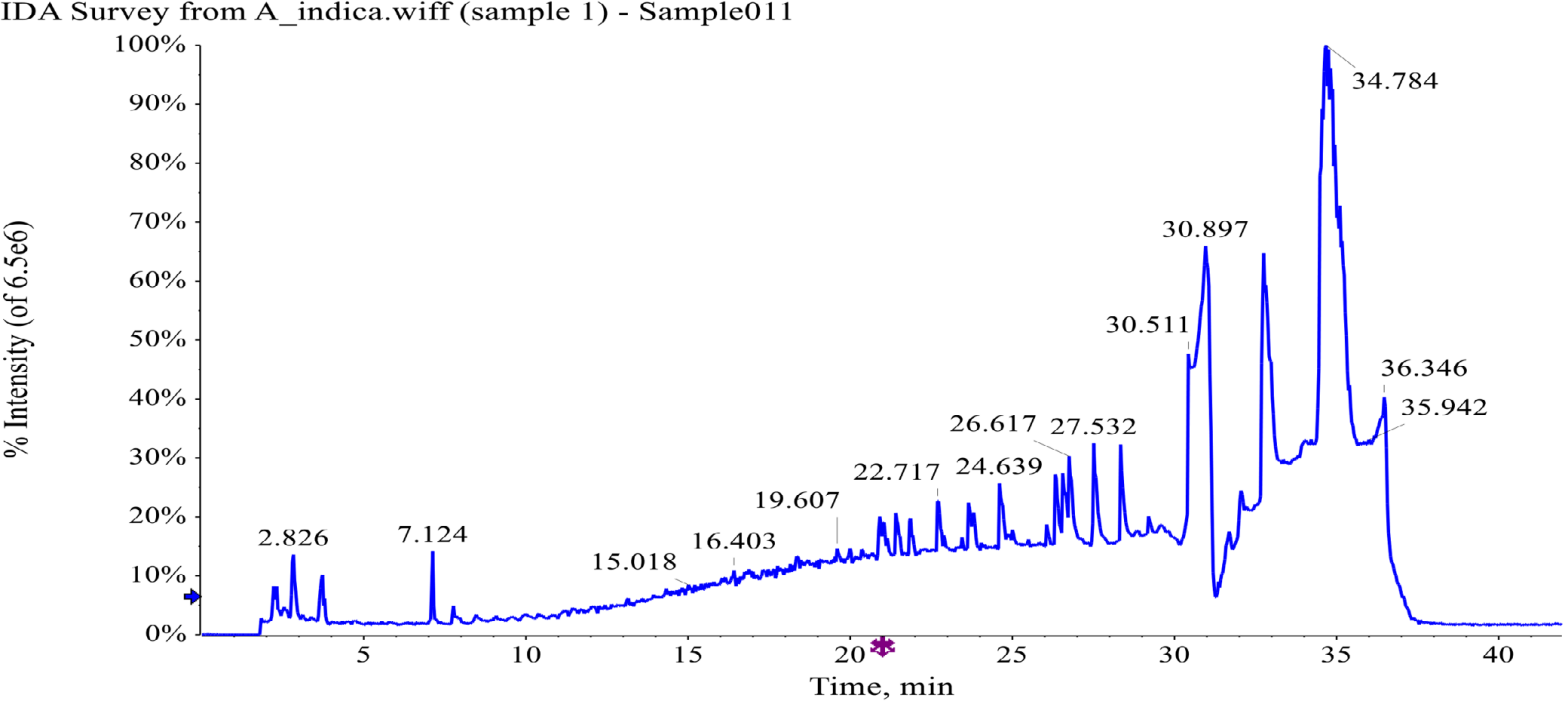
LC–MS/MS (ESI‐QTOF) chromatogram of leave extracts of *Aesculus indica*.

Phytochemical analysis of *A. indica* extract through HPLC‐PDA analysis led to the detection as well as quantification of 10 compounds. These compounds were further examined through LC–MS/MS (Q‐TOF) and findings were confirmed through literature as well as NIST library of which detailed results are compiled (Table 2). A total ion current (TIC)‐based chromatogram of leave extract of *A. indica* is shown in Figure 2, and prominent peaks were probed for the precursor ion of each peak as well as fragmentation pattern (MS/MS) that were compared with the NIST library and reported literature which led to identification of almost 36 compounds mostly phenolics in nature, and details are described in Table 2. The findings of MS study showed that some of the compounds are of phenolics that were identified through HPLC like gallic acid, coumaric acid and their derivatives and some were different in nature which justifies the employment of LC–MS while exploring phytochemical profile of plant extracts. The MS/MS spectrums of all the prominent peaks are presented in supporting information.

**TABLE 2:** Liquid chromatography mass spectrometric (LC‐ESI‐Q‐TOF‐MS/MS) characterization of phytochemicals of *Aesculus indica*.

Sr. #	m/z	RT	MS/MS	Compound name	Reference	Level
1	283.2	20.995	267.1149, 283.1456	Methoxy chrysin	Yasir, Sultana, and Anwar (2018)	2
2	307.2	20.997	247.0883, 283.1241, 267.1141	Pentahydroxyisoflavone	Subbiah et al. (2020)	2
3	325.2	20.998	193.0803, 307.1491, 325.1557	Feruloyl tartaric acid	Ali et al. (2021), Chou et al. (2021)	2
4	339.1	21.000	267.1139, 283.1467, 307.1376, 325.1201	3‐p‐Coumaroylquinic acid	Chou et al. (2021), Wang, He, Li, Lin, et al. (2021)	2
5	355.2	21.002	139.0061, 267.1201, 325.1607, 339.1016	Ferulic acid 4‐O‐glucoside	Chou et al. (2021), Wang, He, Li, Lin, et al. (2021)	2
6	299.2	21.024	237.1024, 279.1178, 295.1474	Enterolactone	Ali et al. (2021)	2
7	323.1	21.026	123.0771, 295.1086, 323.1413	3‐O‐Methylviolanone	Subbiah et al. (2020)	2
8	341.2	21.029	193.0801, 295.1459, 325.1570, 341.1701	Caffeoyl glucose	Ali et al. (2021)	2
9	351.2	21.064	279.1591, 293.1324, 337.1543, 339.1716	Sesamin	Ali et al. (2021)	2
10	169.1	21.082	123.0757, 169.0804	Gallic acid	Pubchem (NIH), NIST, Ali et al. (2021), Subbiah et al. (2020)	1
11	355.2	21.128	123.0734, 295.1139, 309.1630, 323.1416	Pinoresinol	Subbiah et al. (2020)	2
12	214.1	21.216	140.9951, 158.0229, 214.0799	N‐Butylbenzenesulfonamide	Radjai (2021)	2
13	435.3	27.571	131.0768, 423.2312, 425.2222	Quercetin 3‐ arabinoside	Wang, He, Li, Lin, et al. (2021)	2
14	309.2	30.513	284.3258	Dihydroquercitin	Subbiah et al. (2020)	2
15	378.2	30.523	59.0437, 333.2402, 340.3886, 351.1949	7‐Oxomatairesinol	Ali et al. (2021), Chou et al. (2021)	2
16	313.2	30.538	135.1115, 149.1269, 235.1619	Protocatechuic acid 4‐O‐glucoside	Subbiah et al. (2020)	2
17	328.3	30.540	75.0215, 117.0677, 286.2154	Carnosic acid	Ali et al. (2021), Chou et al. (2021)	2
18	383.2	30.551	123.0749, 137.0531, 368.4217, 369.4237	Schisandrin C	Chou et al. (2021), Subbiah et al. (2020)	2
19	418.2	30.555	123.0752, 177.0894, 369.4242, 383.2365	Deoxyschisandrin	Ali et al. (2021)	2
20	253.2	30.570	123.0756, 133.0974	2‐Dehydro‐O‐desmethylangolensin	Subbiah et al. (2020)	2
21	297.2	30.575	284.3268	Sativanone	Ali et al. (2021), Chou et al. (2021)	2
22	307.2	30.576	123.0758, 284.3243, 299.0541	Epigallocatechin	Taslimi et al. (2020)	2
23	325.2	30.580	121.0973, 123.0760, 169.0790, 307.2218, 311.2146	p‐Coumaric acid 4‐O‐glucoside	Ali et al. (2021), Chou et al. (2021)	2
24	369.3	30.586	295.2198, 369.4232	3‐O‐Feruloylquinic Acid	Ali et al. (2021)	2
25	293.2	30.609	284.3262	Catechin	Wang, He, Li, Lin, et al. (2021)	2
26	139.1	30.701	121.0966, 139.1069	Hydroxybenzoic acid	Pubchem(NIH), NIST, Chou et al. (2021), Wang, He, Li, and Wang (2021)	1
27	353.2	32.637	341.2608, 353	3‐Chlorogenic acid	Wang, He, Li, and Wang (2021)	2
28	319.3	32.659	89.0555, 133.0816, 151.0909, 319.1819	Protocatechuic acid 4‐O‐glucoside	Ali et al. (2021), Subbiah et al. (2020), Yasir et al. (2016)	2
29	363.3	32.666	133.0806, 195.1187, 341.2626, 351.1718	Glycosyringic acid	Yasir, Sultana, and Amicucci (2016), Yasir, Sultana, Nigam, et al. (2016)	2
30	554.5	34.643	133.0820, 299.0580, 301.0533, 541.1164	Quercetin 3‐O‐(6‐malonyl glucoside)	Subbiah et al. (2020)	2
31	445.1	34.698	149.0395, 397.1840, 425.2240	Artomunoxanthentrione	Radjai (2021)	2
32	466.4	34.700	133.0808, 359.0272, 397.1768, 425.2097	Chrysoeriol 7‐O‐glucoside (427)	Chou et al. (2021), Yasir, Sultana, and Amicucci (2016)	2
33	515.4	34.768	133.0798, 237.1064, 427.3338, 515.3884	3,4‐O‐Dicaffeoylquinic acid (135)	Yasir, Sultana, and Amicucci (2016)	3
34	537.3	34.770	281.0467, 147.0611, 282.0472, 416.0339	Schisantherin A	Subbiah et al. (2020)	2
35	334.2	34.785	122.0914	Gallic acid‐4‐O‐glucoside	Chou et al. (2021), Subbiah et al. (2020)	3
36	367.2	34.821	237.1068, 323.1419, 367.1684	3‐Feruloylquinic acid	Wang, He, Li, Lin, et al. (2021)	2

*Note:* Mass spectrometry was conducted on negative ion mode.

### 3.2 Effect of *A. indica* Extract on Cell Viability

**FIGURE 3:**
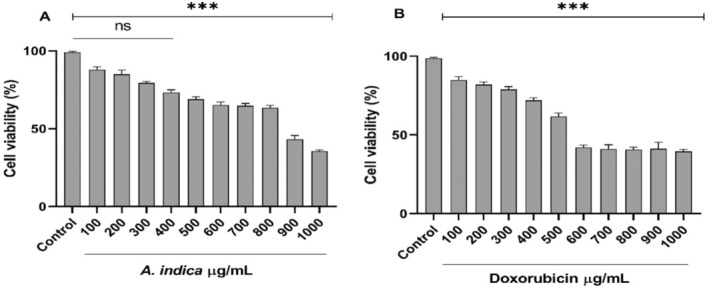
Percentage viability of PBMCs treated with different concentrations (100–1000 μg/mL) *Aesculus indica* (A) and standard Doxorubicin (B) after 72 h. *** indicates *p* < 0.001.

After treatment of PBMCs with *A. indica* extract for 24 h, PBMCs showed a concentration‐dependent reduction in cell viability with IC_50_ value of 783.6 ± 4.38, in comparison to Doxorubicin (IC_50_639 ± 5.84). The extract showed no significant effects on the cell viability on PBMCs up to 100–400 μg/mL (Figure 3), but cell viability decreased slightly at a concentration of 500 μg/mL and at concentrations above 500 μg/mL, the cell viability was significantly decreased as compared to untreated cells.

### 3.3 In Vitro Anti‐Inflammatory Potential of *A. indica*

#### 3.3.1 Effect of *A. indica* Extracts on Pro‐Inflammatory Cytokines Production in PBMCs and WBA

**FIGURE 4:**
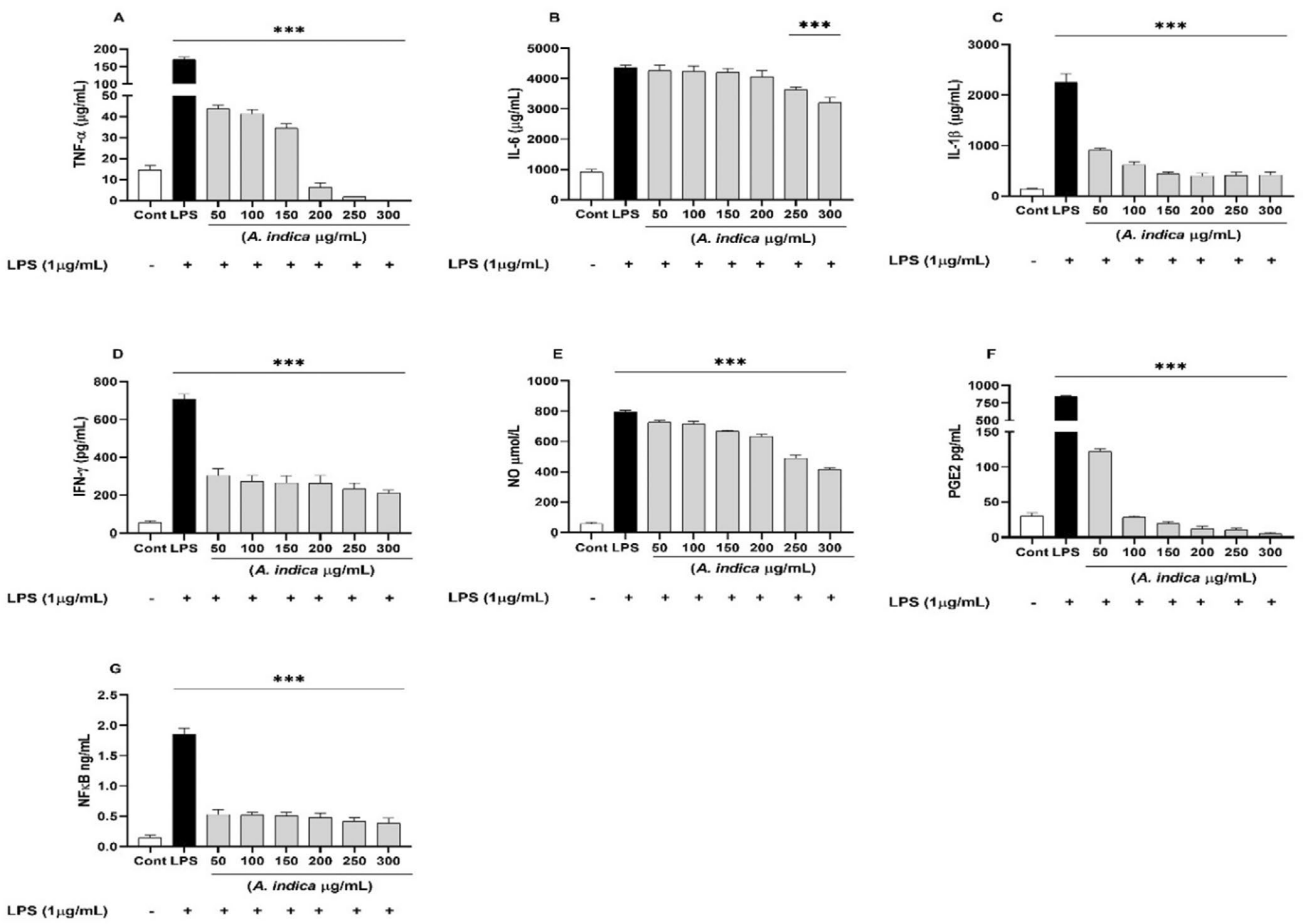
The effect of *Aesculus indica* extract on proinflammatory cytokines (A) TNF‐α, (B) IL‐6, (C) IL‐1β, (D) IFN‐γ, (E) NO, (F) PGE2 and (G) NF‐κB production in LPS‐stimulated PBMCs. The results are presented as mean ± SD (standard deviation). *** indicates *p* < 0.001.

**FIGURE 5:**
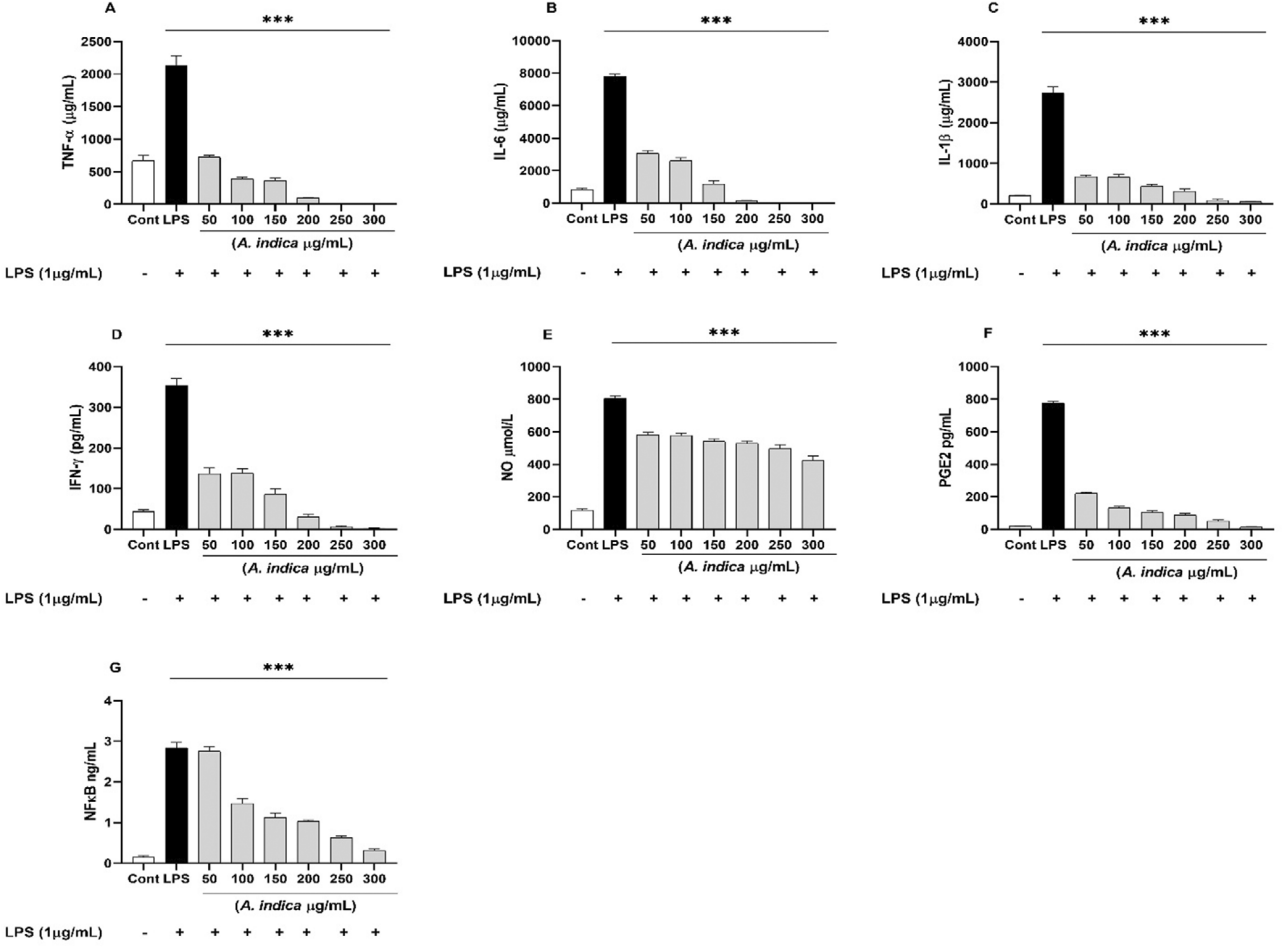
The effect of *Aesculus indica* extract on proinflammatory cytokines (A) TNF‐α, (B) IL‐6, (C) IL‐1β, (D) IFN‐γ, (E) NO, (F) PGE2 and (G) NF‐κB production in LPS‐stimulated WBA. The results are presented as mean ± SD (standard deviation). *** indicates *p* < 0.001.

The levels of four different proinflammatory cytokines (TNF‐α, IL‐1β, IL‐6 and IFN‐γ) were quantified in supernatant of both PBMCs and WBA. Both PBMCs and WBA, when treated with LPS showed increased (*p* < 0.01) production of all four proinflammatory cytokines. However, induction of these cytokines was reversed by hydroethanolic extracts of *A. indica* in dose‐dependent manner (Figures 4A–D and 5A–D). In PBMCs stimulation assay, *A. indica* showed strong inhibition of TNF‐α with IC_50_ values of 21.01 and of 33.72 μg/mL in WBA, respectively (Table 3). TNF‐*α* is a primary proinflammatory cytokine, released after an inflammatory stimulus and eliciting several intracellular events causing NF‐*κ*B activation and leading to production of chemokines proteases and other proinflammatory cytokines (Razmpoosh et al. 2019). Overproduction of cytokines is directly linked with different inflammatory diseases especially rheumatoid arthritis (Ondua et al. 2019). Similarly, LPS treatment increased the IL‐1βlevel in both WBA and PBMCs supernatants. However, *A. indica* extract reduced the levels of IL‐1β in a dose‐dependent manner in WBA, while in PBMCs the effect was not dose dependent, as at 150 μg/mL and above, the effect of the *A. indica* extract on IL‐β is consistent, and not influenced much by increasing concentration. IC_50_ values of PBMCs and WBA for IL‐1β are 138.9 and 16.05 μg/mL, respectively (Table 3). IL‐1β is a powerful inflammatory cytokine involved in several important cellular functions, such as differentiation, activation, and proliferation, and is key component of innate immune response (Chen et al. 2022). IL‐1β also induces the chemotaxis of white blood cells by inducing the IL‐8 induction and activating neutrophils for oxidative burst activity, degranulation, and phagocytosis (Kaplanov et al. 2019). Significant changes in levels of IL‐1β and TNF‐*α* were observed in PBMCs and WBA after treatment with different concentrations of hydroethanolic extract of *A. indica* .

**TABLE 3:** IC_50_ values of *Aesculus indica* extract for TNF‐α, IL‐6, IL‐1β, IFN‐γ, NO, NF‐κB, PGE2 inhibition in LPS‐stimulated PBMCs and WBA.

Sample	PBMCs		WBA	
	*A. indica*	Dexamethasone	*A. indica*	Dexamethasone
TNF‐α	21.01 ± 1.67	23.2 ± 22.75	33.72 ± 3.6	2.66 ± 0.47
IL‐6	413 ± 4.32	258.6 ± 6.81	42.45 ± 3.82	13.38 ± 2.51
IL‐1β	138.9 ± 3.81	1.082 ± 0.07	16.05 ± 1.14	6.627 ± 0.64
IFN‐γ	18.96 ± 3.62	6.218 ± 0.87	8.789 ± 1.55	0.274 ± 0.08
NO	322.7 ± 9.34	298.6 ± 5.31	625.4 ± 8.23	116.2 ± 12.58
PGE2	3.52 ± 0.96	45.78 ± 5.06	14.71 ± 4.33	13.67 ± 3.82
NF‐κB	0.758 ± 0.21	54.96 ± 2.98	126.6 ± 4.60	0.231 ± 0.03

*Note:* Values are expressed as mean ± SD.

Similarly, the *A. indica* extract inhibited the IL‐6 also in a dose‐dependent manner but this response was more obvious in WBA. In PBMCs, the level of IL‐6 was not significantly different from control treated with LPS only. The inhibitory effects of *A. indica* extract on IL‐6 inhibition were significant only at higher concentrations (250‐300 μg/mL) while in WBA, the extract inhibited the IL‐6 in dose‐dependent manner at each level of plant extract up to 100% inhibition of IL‐6 at the highest tested concentration (300 μg/mL). Similarly, IFN‐γ inhibition was not dose dependent in case of PBMCs, while in WBA a significant (*p* < 0.01) dose‐dependent response was observed.IL‐6 is a multifunctional cytokine produced and released by variety of cells, including macrophages, in response to immunological changes, tissue damage and infection, playing a significant role in the regulation of inflammation, hematopoiesis, acute phase reaction immune responses (Jarlborg and Gabay 2022). IL‐6 acts both at systemic and local levels affecting the functionality of different cell types, including not only immune system cells, but also adipocytes, skeletal muscle cells, and hepatocytes (Naseri, Kalantar, and Amirghofran 2018). The hydroethanolic extract of *A. indica* inhibited the IL‐6 in dose‐dependent manner but this response is more obvious in WBA, in PBMCs, level of IL‐6 is not significantly different from control treated with LPS only. IFN‐ γ is a proinflammatory cytokine that is known to play a key role in inflammatory processes and autoimmune disorders. Nowadays, there is developing evidence signifying that IFN‐γ holds surprising potential as a key modulator of inflammation and immune response (Cholet et al. 2019; Zhu et al. 2019). Medicinal plants as one of the most useful natural resources have significant anti‐inflammatory characteristics and help to maintain the function of the immune system. Inhibition of IFN‐γ by plant extracts explored the possible role of anti‐inflammatory action of plant extracts and their constituents.

#### 3.3.2 Effect of *A. indica* Extract on NO and PGE2 Production

The effect of the *A. indica* extract on NO production in LPS‐stimulated PBMCs and WBA was determined by Griess reagent system. LPS significantly (*p* < 0.01) increased the production of NO in both PBMCs and WBA, as compared to untreated cells (Figures 4E and 5E). Extract showed a dose‐dependent inhibition of NO production in both models, but the effect was more obvious in LPS‐stimulated PBMCs as compared to WBA. Similarly, the PGE2 production was significantly (*p* < 0.01) increased by LPS stimulation. Compared with the LPS‐treated group, *A. indica* extract suppressed the secretion of PGE2 in LPS‐stimulated WBA and PBMCs. Moreover, inhibition of PGE2 production levels by *A. indica* extract is comparable to Dexamethasone (Figures 4F and 5F). PGE2 is the most plenteous prostanoid in the human body. Depending upon the situation, PGE2 exerts inflammatory, homeostatic, or in fewer cases anti‐inflammatory effects. PGE2 is a lipid mediator which significantly contributes to the pathogenesis of several inflammatory diseases (Elias et al. 2019; Saengkhae et al. 2021). We found that treatment with plant extracts at higher concentrations significantly suppressed the LPS‐induced PGE2 production. Cells did not produce PGE2 following treatment with *A. indica* extract alone, as there was no difference between plant extracts treated group and LPS‐induced control. During the inflammatory process, NO is sustained and released at very high levels, leading to inflammation of the gut, lung and joints (Adebayo et al. 2019). The synthesis and release of NO encourages inflammation; hence, our findings suggest that *A. indica* extracts may act as inhibitor of its production or scavengers of NO, particularly with corresponding decreased cytotoxicity, could be used to alleviate the propagation of inflammation by NO (Hellal et al. 2020).

#### 3.3.3 Effect of *A. indica* Extracts on NF‐κB

*Aesculus indica* significantly inhibited the NF‐κB level in WBA as well as in PBMCs. The *A. indica* extract showed strongest inhibition of NF‐κB in dose‐dependent manner against both PBMCs and WBA (Figures 4G and 5G). The NF‐κB is an important regulator of inflammatory processes and regulate several aspects of adaptive and innate immune functions. NF‐κB stimulates the expression of several proinflammatory genes encoding chemokines and cytokines which participate in inflammatory reactions. Additionally, NF‐κB plays a major role in differentiation, activation and survival of inflammatory T‐cells and innate immune cells (Yang, Liu, et al. 2021; Yang, Wang, et al. 2021). Therefore, a better understanding of underlying mechanisms of NF‐κB proinflammatory function and activation is of great importance for therapeutic approaches for the treatment of inflammatory conditions.

### 3.4 Assessment of Acute Toxicity and Subacute Toxicity

The acute toxicological study of *A. indica* extract in rats did not exhibit any signs or symptoms in rats treated with a dose of 2000 mg/kg. No change was observed in behavioral, neuronal, and motor functions of animals after administration of single dose for a period of 14 days. The behavioral pattern (posture and gait), activities related to central and autonomic nervous system, eyes, skin and fur examination of experimental groups did not exhibit any changes as compared to vehicle group. Moreover, the body weight of animals, water, and food intake also normal after treatment with *A. indica* extract. Similarly, in subacute toxicological study, the *A indica* extract did not cause any adverse consequences in experimental animals when administered daily for 28 days. Table 4 reveals the change in body weight of animals after treatment with different doses of *A. indica* extract. Repeated dose administration of animals with *A. indica* extract over 28 days showed no significant (*p* > 0.05) variations in body weight in comparison to vehicle group. Moreover, no significant variation (*p* > 0.05) was found in water and food intake of animals in both the groups. After 28 days, the rats were sacrificed and no significant change (*p* > 0.05) in organ weights (liver and kidneys) were recorded in treatment group as compared to vehicle group (Table 5). Our findings suggest that *A. indica* extracts showed no negative effects on animal health, growth, or metabolism.

**TABLE 4:** Effect of *Aesculus indica* extracts on body weights of rats.

Sex	Treatment	Time				
		Day 1	Day 7	Day 14	Day 21	Day 28
Male	Control	212.17 ± 2.19	223.14 ± 3.32	243.17 ± 3.29	289.10 ± 1.36	314.13 ± 1.65
	*A. indica*	219.67 ± 6.39	247.52 ± 3.14	259.18 ± 2.79	275.23 ± 1.64	298.20 ± 2.80
Female	Control	208.22 ± 1.75	226.59 ± 2.21	242.16 ± 2.65	287.19 ± 2.31	319.10 ± 0.81
	*A. indica*	220.76 ± 3.37	251.43 ± 2.58	249.21 ± 5.62	271.32 ± 2.63	289.19 ± 3.71

*Note:* Values are expressed as mean ± SD.

**TABLE 5:** Organ weight of animals (g/g BW) after *Aesculus indica* extract treatment for 28 days.

Sex	Organ	Control	*A. indica*
Male	Liver	2.33 ± 0.22	2.15 ± 0.24
	Kidney	0.57 ± 0.09	0.56 ± 0.05
Female	Liver	2.09 ± 0.06	2.20 ± 0.25
	Kidney	0.56 ± 0.09	0.53 ± 0.05

*Note:* Values are expressed as mean ± SD.

#### 3.4.1 Effect of *A. indica* Extract on Hematological and Biochemical Parameters

Results of hematological parameters of animals after treatment with *A. indica* extract are presented in Table 6. A slight increase in total leukocyte and RBCs count, Hb, packed cell volume was studied in rats of plant treatment groups in comparison to normal control group. However, these differences were not very high and are within the normal ranges of the species. No differences in monocyte, eosinophil and platelets count were observed in both treatment and vehicle groups. Table 7 also showed the tabulated findings of the biochemical evaluation in different animal groups treated with *A. indica* extract. Treatment of rats with *A. indica* did not cause significant variation in serum blood urea nitrogen (BUN), creatinine, or other parameters compared to control group, indicating no important (*p* > 0.05) variations in kidney function after treatment with *A. indica* extract.

**TABLE 6:** Hematological profile of rats after treatment with *Aesculus indica* extract for 28 days.

Parameter	Control	*A. indica*
Hemoglobin (g/dL)	14.88 ± 2.00	15.02 ± 1.44
Packed cell volume	44 ± 1.55	34 ± 1.91
Red blood corpuscles (10^12^/L)	6.90 ± 0.35	5.5 ± 0.93
MCV (fl)	57 ± 3.13	61 ± 1.65
MCH (g/L)	23 ± 2.54	22 ± 3.62
MCHC (pg)	36 ± 1.52	36 ± 1.61
White blood cells (10^9^/L)	7.8 ± 0.31	14.3 ± 0.63
Platelets (10^9^/L)	164 ± 2.43	1186 ± 77.01
Neutrophils (%)	10 ± 0.66	31 ± 2.33
Lymphocytes (%)	83 ± 2.54	51 ± 1.64
Monocytes (%)	3 ± 0.24	10 ± 0.67
Eosinophils (%)	4 ± 0.68	8 ± 0.77

*Note:* Values are expressed as mean ± SD.

**TABLE 7:** Biochemical profile of rats after treatment with *Aesculus indica* extract for 28 days.

Parameter	Control	*A. indica*
AST (U/L)	32 ± 1.41	29 ± 1.62
ALT (U/L)	35 ± 1.32	32 ± 4.02
ALP (U/L)	451 ± 3.33	595 ± 5.63
ɣ‐GT (U/L)	6.69 ± 0.90	4.90 ± 0.92
T. BILL (μmol/L)	0.6 ± 0.01	0.6 ± 0.01
D. BILL (μmol/L)	0.2 ± 0.03	0.2 ± 0.05
I. BILL (μmol/L)	0.4 ± 0.04	0.4 ± 0.11
Glucose (mmol/L)	5.00 ± 1.49	5.01 ± 0.21
Creatinine (μmol/L)	63.11 ± 2.87	66.42 ± 3.33
T. cholesterol (mmol/L)	1.68 ± 0.16	1.73 ± 0.11
Triglycerides (mmol/L)	0.91 ± 0.02	1.02 ± 0.10
Total protein (g/L)	4.39 ± 0.07	5.32 ± 0.92
BUN (mmol/L)	9.01 ± 0.34	6.02 ± 1.93

*Note:* Values are expressed as mean ± SD.

The results of biochemical parameters of treatment groups did not significantly differ from the untreated group and the results exclude any toxic properties of the plant extracts on liver. When exposed to toxic substances, the kidney and liver exhibit toxic characteristics since they are both crucial to the detoxification process. Treatment of rats with *A. indica* extract did not change biochemical parameters, suggesting no considerable changes in the kidney and liver function after *A. indica* extract treatment. The hematopoietic system is also an important standard of pathological and physiological status of animals (Ghauri et al. 2022). The histopathological portion of the acute toxicity study paralleled the findings of liver and kidney parameters (Ekanayake et al. 2019). In contrast to untreated group, appearance of hematoxylin‐ and eosin‐stained tissue parts of treatment rats appeared normal in structure after treatment of rats with *A. indica* extracts under light microscope. The histological evaluation plays a significant part in the assessment of organ damage resulting from treatments as biopsy can provide valuable evidence by the removal of opposing causes of tissue damage (Liyanagamage et al. 2020). The findings showed that regular administration of the *A. indica* extracts up to 28 days did not produce any morphological disturbances or harmful changes to the rats or to the vital organs.

### 3.5 Paw Inflammation Model

The results of in vivo anti‐inflammatory activity of *A. indica* extract are given in Table 8. Results indicate that *A. indica* extract showed significant (*p* < 0.01) inhibition of paw edema in time and dose‐dependent manner. The maximum inhibition percentage of inflammation was observed after 5th hour compared to control group. The percentages of inflammation and inhibition (PI) in the 2nd hour was, 73.93% in *A. indica* treated group at 400 mg/kg BW and 71.30% in the reference group (dexamethasone 20 mg/kg), respectively. By comparison, after 5 h, the PI was 74.99% in *A. indica* treated group (400 mg/kg BW) group and 85.34% in the reference group, respectively (Table 8). The carrageenan‐induced rat paw edema model was commonly used to evaluate the anti‐inflammatory properties of natural and synthetic compounds. Carrageenan‐induced rat paw edema model is biphasic phenomenon in nature (Rafiee, Hajhashemi, and Javanmard 2019). After 1–2 h of carrageenan injection (first phase) is mediated by bradykinins, serotonin and histamine which are secreted into the surrounding damaged tissues from mast cells. After 3–6 h (second phase) of carrageenan injection, the inflammatory reaction is correlated with the release of arachidonate metabolites such as leukotrienes, prostaglandins and different cytokines (Fitri et al. 2021). Injection of carrageenan in the paw triggered a vascular phase of inflammation characterized by vasodilation resulting in increased inflammation in all groups (Faisal et al. 2022). Carrageenan injection caused intense inflammation that peaked after 3 h. Our findings suggest that *A. indica* has antagonistic effects on many inflammatory mediators involved in cellular and vascular stages.

**TABLE 8:** Effects of different concentration of *Aesculus indica* extract on paw inflammation in rats.

Time	Control	Carrageenan	*A. indica* (mg/Kg)			Dexamethasone 20 (mg/Kg)
			100	200	400	
0 h	0.51 ± 0.01	0.32 ± 0.03	18.53 ± 1.23	41.40 ± 2.93	72.18 ± 2.27	70.35 ± 1.03
1 h	0.55 ± 0.04	0.45 ± 0.02	19.34 ± 1.66	41.81 ± 0.89	73.57 ± 0.65	72.19 ± 1.91
2 h	1.32 ± 0.06	0.29 ± 0.05	19.42 ± 150	43.61 ± 1.18	73.93 ± 0.37	72.10 ± 1.00
3 h	1.39 ± 0.03	0.23 ± 0.02	20.10 ± 2.59	44.29 ± 2.05	74.31 ± 0.65	72.55 ± 1.22
4 h	1.85 ± 0.05	0.22 ± 0.04	21.81 ± 2.88	44.73 ± 1.55	74.63 ± 1.12	74.19 ± 2.14
5 h	1.92 ± 0.03	0.15 ± 0.01	23.49 ± 1.17	45.23 ± 0.71	74.99 ± 1.69	76.24 ± 1.07

*Note:* Values are presented mean ± SD of three replicates.

#### 3.5.1 Histopathological Examination of Paw Tissues

**FIGURE 6:**
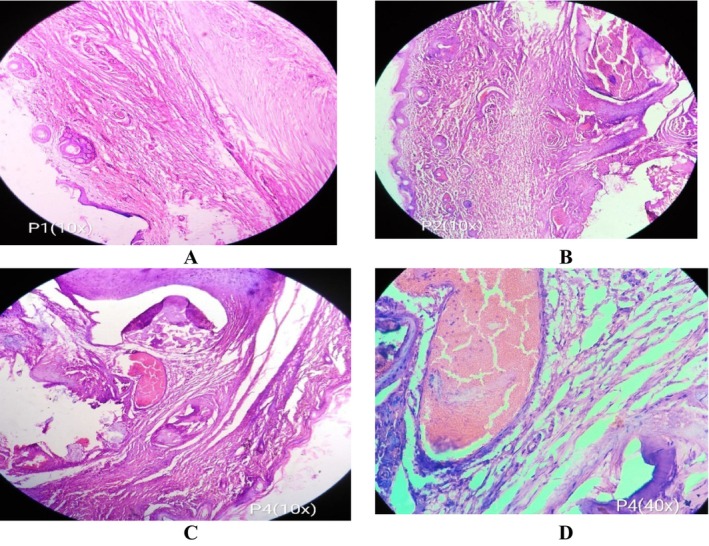
Representative photographs from rat paw showing the protective effect of *Aesculus indica* extract against carrageenan‐induced paw inflammation in rats, (A) Dexamethasone (B, C), rats treated with carrageenan plus *A. indica* (D) Untreated control.

Results of histopathological observations of paw tissue biopsies are shown in Figure 6. A microscopic view of paw biopsies of disease control groups revealed significant cellular infiltration in the connective tissue of both dermis and epidermis with acute edema. Similarly, presence of subcutaneous edema with infiltration of inflammatory cells, especially polynuclear neutrophils at the inflamed tissue site, and a spongy appearance of the epidermis was also observed in carrageenan‐treated paw. A decrease in cellular infiltration as well as decrease in spongy like appearance was observed in biopsies of animals treated with dexamethasone and *A. indica* extract. Moreover, the number of inflammatory cells was reduced and confined to the vicinity of the vascular area in comparison to disease control (carrageenan‐treated) group.

#### 3.5.2 Effect on Lipid Peroxidation, Enzymatic Antioxidant Status and Oxidative Stress Parameters in Rat Paw Tissues

**FIGURE 7:**
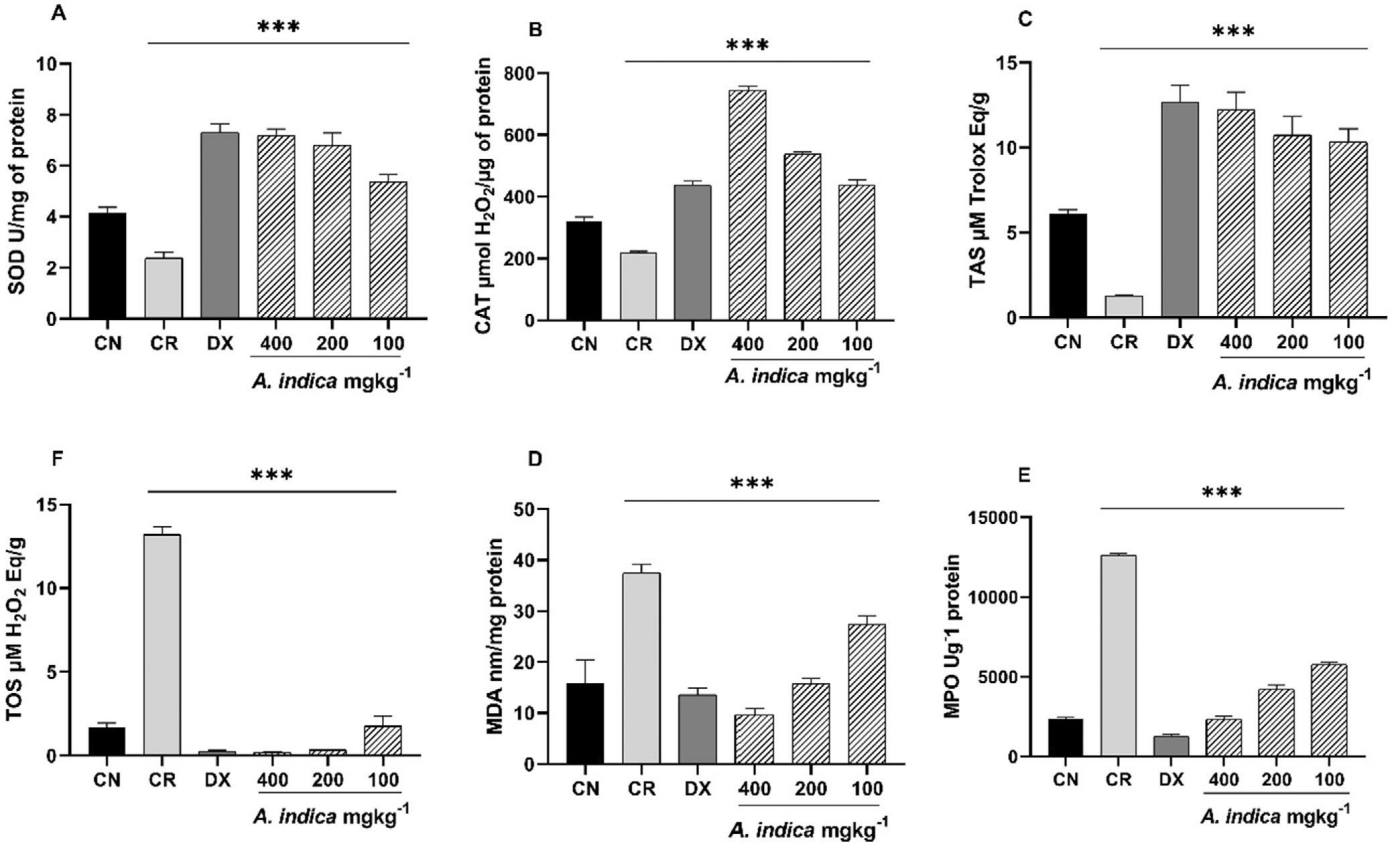
The effect of *Aesculus indica* extract on (A) SOD, (B) CAT, (C) TAS, (D) TOS, (E) MPO and (F) MDA level in paw tissue homogenate. The results are presented as mean ± SD (standard deviation). *** indicates *p* < 0.001.

To evaluate the role of lipid peroxidation as marker of oxidative stress, MDA level was measured in all treated groups, and it was noted that level of MDA increased significantly (*p* < 0.001) after injection of carrageenan (carrageenan group) and reduced significantly in all groups treated with different concentration of plant extracts (Figure 7). Dexamethasone decreased the MDA and MPO level significantly as compared to all *A. indica* treated group. Furthermore, treatment with *A. indica* extracts also prevented the increased MPO activity in dose‐dependent manner but results were more pronounced in reference drug group.

The obtained results of CAT and SOD levels in the paw tissues of the different studied groups are summarized in Figure 7. Induction of inflammation through carrageenan injection led to a significant decrease in both antioxidant enzymes (SOD and CAT) as compared to normal control group (*p* < 0.001). However, pre‐treatment of rats with hydroethanolic extracts of *A. indica* restored the tissue enzymatic activity significantly. Dexamethasone as reference drug showed the highest reversal of enzymatic activity caused by carrageenan injection. Level of TAS is therefore increased in treated groups due to increase in SOD and CAT activity and decrease of TOS level in all tissues. Tissue MPO, lipid peroxidation through MDA, SOD, CAT along with TAS and TOS in the rat paw tissues were measured at 5 h of carrageenan induction and it was observed that level of these biochemical markers altered significantly after subcutaneous injection of carrageenan (5 h) compared to non‐treated tissues of rats. Induction of inflammation through carrageenan injection led to a significant decrease in both antioxidant enzymes (SOD and CAT) as compared to normal control group. However, pre‐treatment of rats with hydroethanolic extract of *A. indica* restored the tissue enzymatic activity significantly. Dexamethasone as reference drug showed the highest reversal of enzymatic activity caused by carrageenan injection. Level of TAS is therefore increased in treated groups due to increase in SOD and CAT activity and decrease of TOS level in all tissues.

## 4 Conclusion

On the basis of current findings, we suggest that *A. indica* extract is a potential source of bioactive constituents as characterized through HPLC‐PDA and LC–MS/MS. These bioactive fingerprints of *A. indica* possess potential anti‐inflammatory activities. The proposed mechanism of these bioactive fingerprints is the downregulation of NF‐κB and TNF‐α which further suppress the expression of IL‐6, IL‐1β, NO and PGE2. It is possible to conclude that the *A. indica* extract reduces cellular infiltration, and other inflammatory parameters related to oxidative stress in carrageenan‐induced rat paw edema model. However, more studies are required to explore the effects of these isolated and purified compounds and their mechanisms of action.

## Author Contributions

**Hina Fatima:** data curation (equal), formal analysis (lead), investigation (lead), methodology (lead), software (equal), visualization (equal), writing – original draft (equal). **Muhammad Shahid:** conceptualization (equal), funding acquisition (equal), project administration (lead), resources (equal), supervision (lead), validation (equal), writing – review and editing (supporting). **Sana Fatima:** data curation (supporting), formal analysis (supporting), software (supporting), writing – original draft (equal). **Paul J. Mills:** conceptualization (supporting), funding acquisition (equal), methodology (supporting), project administration (equal), resources (equal), supervision (supporting), validation (equal), visualization (supporting). **Chris Pruitt:** formal analysis (supporting), resources (equal), software (supporting), validation (equal), visualization (supporting), writing – review and editing (supporting). **Meredith A. Pung:** investigation (supporting), project administration (supporting), resources (supporting), software (equal), validation (supporting), visualization (equal). **Muhammad Riaz:** formal analysis (supporting), methodology (supporting), visualization (equal), writing – review and editing (lead). **Rizwan Ashraf:** formal analysis (supporting), software (equal), visualization (equal), writing – review and editing (supporting). **Quzi Sharmin Akter:** software (equal), validation (equal), visualization (supporting), writing – review and editing (equal).

## Conflicts of Interest

The authors declare no conflicts of interest.

## Data Availability

The data will be available from principal and corresponding authors on reasonable request.
